# Neuroprotective Potency of Tofu Bio-Processed Using *Actinomucor elegans* against Hypoxic Injury Induced by Cobalt Chloride in PC12 Cells

**DOI:** 10.3390/molecules26102983

**Published:** 2021-05-18

**Authors:** Liqing Yin, Yongzhu Zhang, Fidelis Azi, Mekonen Tekliye, Jianzhong Zhou, Xiaoli Liu, Mingsheng Dong, Xiudong Xia

**Affiliations:** 1 College of Food Science and Technology, Nanjing Agricultural University, 1 Weigang Road, Nanjing 210095, China; 2016208017@njau.edu.cn (L.Y.); azifideel@yahoo.com (F.A.); cherinet2018@gmail.com (M.T.); zjzluck@126.com (J.Z.); liuxljaas@hotmail.com (X.L.); 2 Institute of Agricultural Product Processing, Jiangsu Academy of Agricultural Sciences, No. 50 Zhongling Street, Nanjing 210014, China; 3 Institute of Food Safety and Nutrition, Jiangsu Academy of Agricultural Sciences, No. 50 Zhongling Street, Nanjing 210014, China; 20200025@jaas.ac.cn; 4 School of Food and Biological Engineering, Jiangsu University, 301 Xuefu Road, Zhenjiang 212013, China

**Keywords:** soybean products, oxidative stress, cell apoptosis, cell arrest, autophagy

## Abstract

Fermented soybean products have attracted great attention due to their health benefits. In the present study, the hypoxia-injured PC12 cells induced by cobalt chloride (CoCl_2_) were used to evaluate the neuroprotective potency of tofu fermented by *Actinomucor elegans* (FT). Results indicated that FT exhibited higher phenolic content and antioxidant activity than tofu. Moreover, most soybean isoflavone glycosides were hydrolyzed into their corresponding aglycones during fermentation. FT demonstrated a significant protective effect on PC12 cells against hypoxic injury by maintaining cell viability, reducing lactic dehydrogenase leakage, and inhibiting oxidative stress. The cell apoptosis was significantly attenuated by the FT through down-regulation of caspase-3, caspases-8, caspase-9, and Bax, and up-regulation of Bcl-2 and Bcl-xL. S-phase cell arrest was significantly inhibited by the FT through increasing cyclin A and decreasing the p21 protein level. Furthermore, treatment with the FT activated autophagy, indicating that autophagy possibly acted as a survival mechanism against CoCl_2_-induced injury. Overall, FT offered a potential protective effect on nerve cells in vitro against hypoxic damage.

## 1. Introduction

Soybean is a crucial crop and has been consumed for thousands of years in Asia. Wide varieties of soy products are available in everyday life, including tofu, soymilk, soy mayonnaise, boiled soybeans, roasted soybean, and textured soy protein, which are popular all over the world due to their high amount of protein, unsaturated fatty acid, minerals, vitamins, and especially isoflavones [1,2,3]. Soybean isoflavones are important phenolic sources in our daily diet [4], and are beneficial to human health due to their high antioxidant activity [2]. Due to the balanced nutritional components, the daily dietary intake of soybean products has been recommended as a healthy approach towards reducing the risks of cardiovascular disease, obesity, cancer, and diabetes [3,4].

In recent years, fermentation has been an efficient approach to improving the nutrition and bioactivity of soybean foods, and their application plays a crucial role in producing biomolecules such as organic acids, phenolic compounds, flavor, and pigments [5]. Shahzad et al. (2020) indicated that soybean fermented with *Bacillus amyloliquefaciens* RWL-1 has a higher content of total amino acids, antioxidants, total phenolic, and isoflavones than that of soybean [6]. Xiao et al. (2014) reported a significant increase in the total phenolics, saponins, protective effects against DNA damage, and antioxidant capacity of chickpeas after fermentation with *Cordyceps militaris* SN-18 [7]. Ali et al. (2017) also found that the total amino acids, isoflavone aglycone, and antioxidant activity of soybean significantly increases after fermentation with probiotic *Bacillus subtilis* [8]. Mao-tofu fermented with *Mucor* spp. is a traditional healthy snack in China, which shows a distinct flavor and cheese-like consistency [9]. Previous studies have reported that *Mucor*-fermented tofu presents remarkable anti-hypertension activity and more evident immune activity than those of unfermented tofu [9,10]. However, little attention is paid to studying the neuroprotective effects of tofu fermented with *A. elegans*.

Studies on degenerative brain diseases have attracted significant attention in recent years due to the potential to prolong lifespan [11]. Reactive oxygen species (ROS) levels play a vital role in the development of neurodegenerative diseases, including Parkinson and Alzheimer Disease, by oxidizing biomacromolecules (DNA, proteins, and lipids), destroying mitochondrial function, and promoting neuronal apoptosis [11]. Therefore, preventing oxidative-stress-induced apoptosis may be a potential therapeutic strategy in treating neurodegenerative diseases [12]. Many studies have been performed to develop healthy and functional foods that help improve memory or cognitive abilities by using natural products, such as *ginkgo* leaf, *evodia* fruit, *angelica* root, and *Codonopsis lanceolata* [13]. In the present study, the neuroprotective effects of fermented tofu with *A. elegans* against hypoxic injury induced by cobalt chloride (CoCl_2_) in PC12 cells were investigated. The efforts were also made to study the effects of fermented tofu on autophagy in PC12.

## 2. Results and Discussion

### 2.1. Phenolic Content and Antioxidant Activity

**Figure 1:**
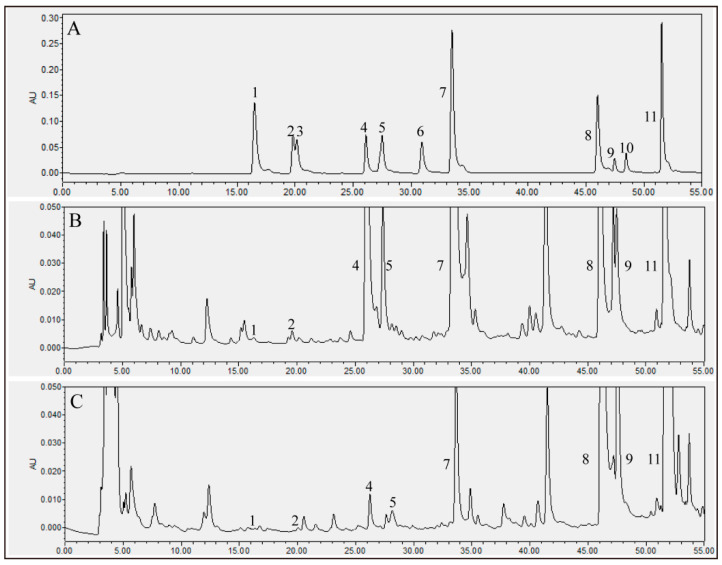
HPLC chromatograms of phenolic content in UT and FT. (**A**) a mixture of 11 standards; (**B**) the phenolics in UT; (**C**) the phenolics in FT. 1, chlorogenic acid; 2, vanillic acid; 3, caffeic acid; 4, daidzin; 5, glycitin; 6, ferulic acid; 7, genistin; 8, daidzein; 9, glycitein; 10, quercetin; 11, genistein. UT, unfermented tofu; FT, fermented tofu.

The phenolic composition of tofu was analyzed using high-performance liquid chromatography (HPLC) (Figure 1). As shown in Table 1, fermentation with *A. elegans* significantly increased the total phenolic content (TPC) of tofu. The TPC in the fermented tofu (FT) extract was 1835.67 mg gallic acid equivalent (GAE)/100 g, which was 1.45 times that in the unfermented tofu (UT) extract. The phenolic compounds in plants commonly occur as insoluble forms by forming a covalent bond with cellular components such as cellulose, proteins, and sugars, resulting in a low bioavailability for humans [14]. The insoluble phenolics can be released by enzymes generated from microorganisms, which could break down the bond between phenolics and cellular components [15]. Bei et al. (2018) showed that oat fermentation by *Monascus anka* significantly increased the phenolic content in correlations with fpase, xylanase, and α-amylase activities [16]. De Camargo et al. (2016) also found that pronase and viscozyme from *Saccharomyces cerevisiae* can efficiently convert insoluble phenolics to soluble forms, thereby increasing the TPC of winemaking by-products [17]. Therefore, the increased TPC in the fermented tofu observed in our study might be due to the enzymes such as α-amylase, β-glucosidase, endoglucanase, exoglucanase, esterase, and protease released by *A. elegans* during fermentation [18].

Soybean isoflavones, including glycitein, glycitin, daidzin, daidzein, genistin, and genistein, were the predominant phenolic components in soybean foods [1]. As shown in Table 1, the total isoflavone content occupied 84.70% of the TPC in UT. The glycoside isoflavones of the soy were efficiently transformed to their corresponding aglycones after *A. elegans* fermentation. The glucoside isoflavones in tofu significantly decreased by 91.10%, while the aglycone isoflavone content showed a significant 1.81 times increase. Results indicated that *A. elegans* fermentation was an effective way to enrich aglycone isoflavones in tofu. The chlorogenic acid and vanillic acid were also identified by HPLC analysis (Figure 1), which showed an obvious decrease after fermentation. This might be because these phenolic compounds were hydrolyzed by the enzyme produced from *A. elegans* during fermentation. Plant polyphenols are potent free-radical scavengers [7,19], and aglycone isoflavones exhibit higher antioxidant activities and are much more easily absorbed by a human than their glucoside forms [20,21]. Hence, a higher antioxidant activity of FT extract was obtained compared with that of UT due to the increased TPC and aglycone isoflavone content. As shown in Table 2, the 2,2-azinobis(3-ethylbenzothiazoline-6-sulfonic acid) diammonium salt (ABTS) radical cation scavenging capacity, reducing power (RP), and ferric reducing antioxidant power (FRAP) of FT were respectively 89.97%, 0.33 (OD_700_), and 643.08 (μM FeSO_4_), which compared to UT were 33.29%, 83.33%, and 68.99% higher, respectively. It has been shown that polyphenols with high antioxidant activity are beneficial for human health to reduce the risks of several diseases such as neurodegenerative disease [22], ischemic injury [23], cancer [24], and hyperlipidemia [25]. Therefore, it was expected that fermentation with *A. elegans* could improve the functional properties of tofu.

### 2.2. Evaluation of CoCl_2_, UT, and FT for Cytotoxicity

**Figure 2:**
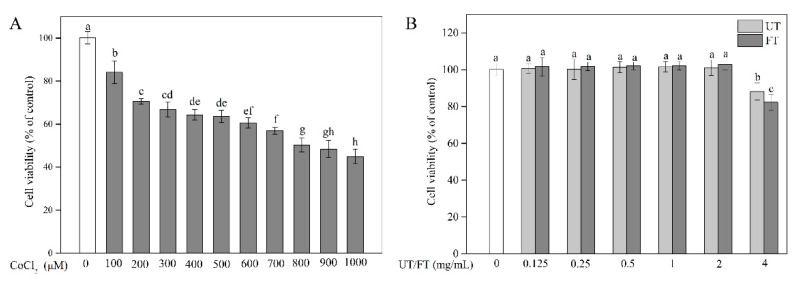
Cytotoxicity evaluation. (**A**) Cytotoxicity of CoCl_2_. (**B**) Cytotoxicity of UT and FT extracts. Means with different small letters were significantly different (*p* < 0.05).

CoCl_2_ is an active inducer commonly used to cause hypoxic/ischemic conditions in vitro. In this study, the cell toxicity induced by CoCl_2_ was assessed by methyl thiazolyl tetrazolium (MTT) assay. An obvious dose-dependent manner was observed in the CoCl_2_-induced injury (Figure 2A). The PC12 cell viability exhibited a rapid decrease upon exposure to CoCl_2_, which demonstrated that CoCl_2_-induced toxicity caused severe damage to PC12 cells. Exposure to 800 μM CoCl_2_ for 12 h resulted in a 49.71% decrease in cell viability compared to the untreated control. Therefore, 800 μM CoCl_2_ was used to cause hypoxic injury in the present study. To investigate the neuroprotective effects of UT and FT against hypoxic injury, the safe doses of UT and FT extracts on PC12 cell viability were also studied (Figure 2B). Treatment with a dosage of 0.125 to 2 mg/mL of the UT or FT extracts for 12 h showed no toxicity on PC12 cells, while using a higher dosage at 4 mg/mL led to a significant decrease in cell viability. This could be due to the generation of ROS during the auto-oxidation of phenolics at higher concentrations of UT and FT [26]. Therefore, the safe dose of UT and FT extract (<4 mg/mL) was used for the following experiments.

### 2.3. Protective Effects of UT and FT against Hypoxic Injury

**Figure 3:**
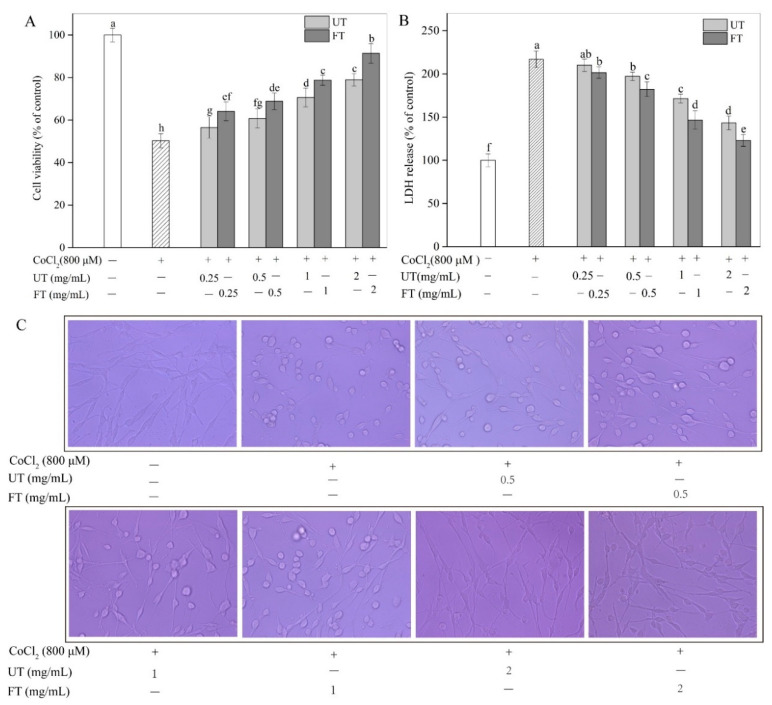
Protective effects of UT and FT extract on CoCl_2_-induced injury. (**A**) Cell viability. (**B**) LDH level. (**C**) Cell morphology. Means with different small letters were significantly different (*p* < 0.05).

The cell viability of PC12 cells was significantly declined due to exposure to 800 μM CoCl_2_ (Figure 3A). However, treatment of the cells with either of UT and FT extracts significantly alleviated the CoCl_2_-induced cell injury in a dose-dependent manner, indicating a protective effect for UT and FT on PC12 cells in hypoxic injury. Using 2 mg/mL FT extract exhibited the highest protective effect on cell viability against the hypoxic injury, increasing the cell viability from 50.29% in the hypoxic control to 91.39% in the FT-treated cells. The results also indicated an enhanced and significantly higher protective effect for the fermented samples by *A. elegans* compared to unfermented samples. It has been shown that hypoxic damage can induce strong oxidative stress along with the overproduction of ROS, which can then oxidize lipids, DNA, and proteins. Such damage to the cells can result in adverse consequences such as interruption of mitochondrial function, activation of pro-apoptosis-related proteins, enhanced neuronal apoptosis, all leading to neurodegenerative diseases [11]. Plant antioxidants such as flavonoids, polyphenols, and anthocyanins, can efficiently scavenge the free radicals produced during cells’ oxidative injury [19,27,28]. Soybean isoflavones with high antioxidant activities exhibit great ability to protect nerve cells against oxidative injury [29]. Consequently, the cytoprotection of UT and FT extracts were rooted in the antioxidant activities of the phenolic compounds presented in the extracts.

CoCl_2_-induced cell injury can also manifest as compromised cell membrane integrity, resulting in lactate dehydrogenase (LDH) leakage [30]. The LDH release was also measured in PC12 cells exposed to different treatments (Figure 3B). The LDH released from PC12 cells was significantly increased by CoCl_2_, which was 2.17-fold of that from untreated control. Interestingly, the LDH level was significantly decreased by UT and FT treatments at concentrations of 0.25 mg/mL to 2 mg/mL in a dose-dependent manner. For example, the LDH level in the UT-treated cells at 2 mg/mL was only 66.07% of that in only CoCl_2_-treated control. The protective effect of tofu was significantly enhanced after *A. elegans* fermentation. For example, PC12 cells treated with 2 mg/mL FT extract showed a much lower LDH leakage, which was only 85.83% of that in the UT extract-treated cells. Furthermore, an obvious improvement in the cellular morphology was observed in a dose-dependent manner when PC12 cells were treated with UT or FT extracts (Figure 3C). Cells incubated with only CoCl_2_ were crimpled into the spherical state, indicating severe damage. However, UT and especially FT treatments retained a much more similar cell morphology compared to that of the control cells (untreated) in which the cells were looking relatively filamentous and elongated. The observed protective effects of the FT treatment on the cell morphology was also greater than the UT treatment.

### 2.4. Attenuation of CoCl_2_-Induced Oxidative Stress with UT and FT

**Figure 4:**
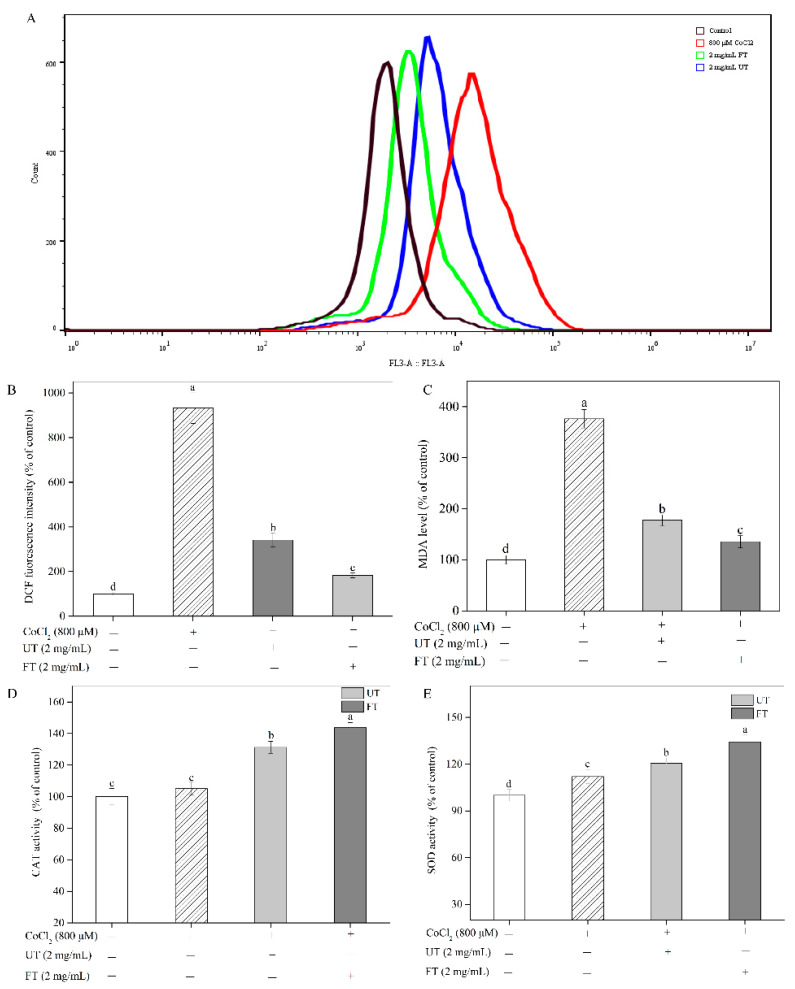
Protective effects of UT and FT extracts on CoCl_2_-induced oxidative stress. (**A**) ROS level. (**B**) The DCF fluorescence intensity measured using a BD Accuri^TM^ C6 Flow cytometer. (**C**) MDA level. (**D**) CAT. (**E**) SOD. White, patterned, light gray, and dark gray indicated untreated control (without CoCl_2_, UT and FT), only CoCl_2_-treated, UT + CoCl_2_-treated, and FT + CoCl_2_-treated group. Significant differences between any two groups above were checked. The small letters (a, b, c, and d) indicated the ordering of highness of each group, since “a” means the highest yield and “d” means the smallest amount. Every group was used as control to compare the difference in this figure. Means with different small letters were significantly different (*p* < 0.05).

Hypoxic damage can induce substantial oxidative stress in cells along with a large amount of ROS formation [31]. Figure 4A,B depict the effect of UT and FT extracts on ROS production induced by CoCl_2_ was also studied in PC12 cells. CoCl_2_-induced toxicity resulted in a significant increase in the ROS level, which was 9.32 times that in the untreated PC12 cells. However, as expected, the ROS generation was significantly inhibited after PC12 cells were treated with the UT extract at 2 mg/mL. The ROS levels decreased from 932.25% in the CoCl_2_-treated cells to 341.55% in UT-treated cells compared to control. Cho et al. (2017) reported that sea buckthorn (*Hippophae rhamnoides* L.) leaves with a high content of phenolic compounds, such as quercetin, gallic acid, and ellagic acid, could significantly attenuate intracellular oxidative stress in PC12 cells [27]. Qian et al. (2012) also demonstrated that a soy isoflavone, genistein, could significantly decrease ischemia-induced ROS production and enhance the antioxidant enzyme activities of SOD and glutathione peroxidase in stroke mice [29]. Hence, the observed reduction in ROS levels in the UT-treated cells might be due to the free radical scavenging activity of phenolics and isoflavones. Similar to the previous observations, using 2 mg/mL FT extract showed a significant enhancement in reducing the intracellular ROS levels to as low as 183.50%, which is about half of the measured values for the UT-treated cells. Excessive ROS generally leads to intracellular lipid peroxidation. As shown in Figure 4C, the toxicity induced by CoCl_2_ in PC12 cells was accompanied by significantly increased lipid peroxides. The malondialdehyde (MDA) level in PC12 cells increased by 2.76 times in the CoCl_2_-treated group compared to control. However, the UT and FT treatments both significantly decreased the MDA level in PC12 cells.

Intracellular antioxidant enzymes, such as catalase (CAT) and superoxide dismutase (SOD), play important roles in the defensive mechanism to prevent ROS oxidative injuries [19]. Compared with untreated cells, CAT and SOD activity of the CoCl_2_-treated cells increased by 5.10% (*p* > 0.05) and 12.02% (*p* < 0.05), respectively (Figure 4D,E), possibly due to the activation of the intracellular adaptive defense mechanism against the overproduction of ROS in PC12 cells, which was in agreement with Zhang et al. (2020) [30] and Anwar et al.’s (2014) observations [32]. However, the expressed enzymatic activity was not enough for cells to offset CoCl_2_-imposed toxicity. This situation was improved by UT and FT treatments at 2 mg/mL, and significant increases in antioxidant enzyme activities of CAT and SOD were obtained in Figure 4D,E. Furthermore, the antioxidant enzymes CAT and SOD in FT-treated cells exhibited the highest activities, which increased by 36.73% and 19.85% compared with those in only CoCl_2_-treated cells, respectively. A higher level of antioxidant enzyme activities contributed to improving the scavenging ability of free radicals of PC12 cells, which explained the reason why a lower ROS level was observed in the FT-treated cells than that in the UT-treated cells.

### 2.5. Retardation of CoCl_2_—Induced Cell Apoptosis by the UT and FT

**Figure 5:**
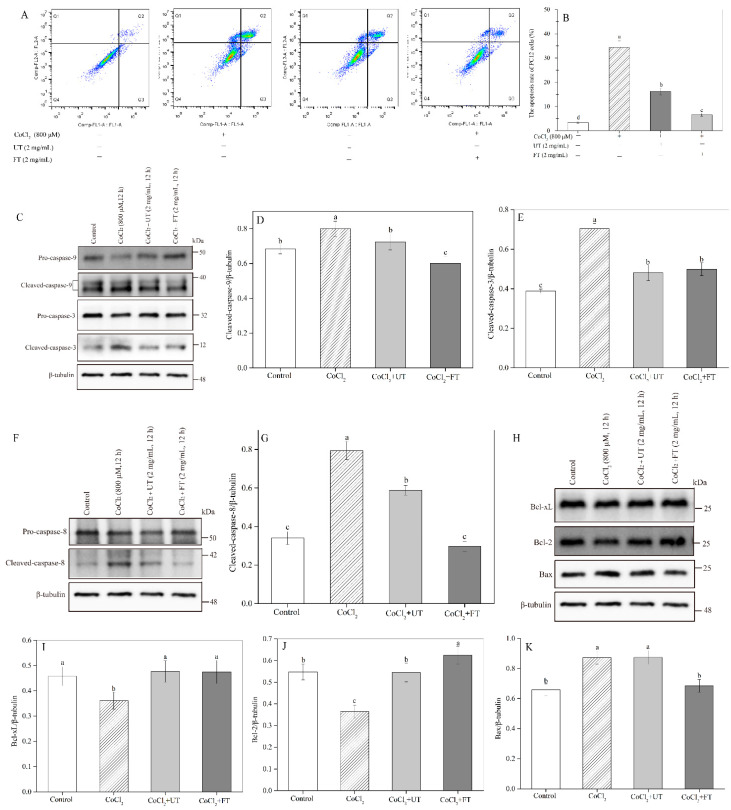
Effects of UT and FT extract on the CoCl_2_-induced cell apoptosis. (**A**) Cell apoptosis. (**B**) The total PC12 cell apoptosis rate. (**C**) The western blot analysis of caspase-3 and caspase-9 protein expression. (**D**,**E**) The densitometric semi-quantifications of the western blots in (**C**). (**F**) The western blot analysis of caspase-8. (**G**) The densitometric semi-quantifications of the western blots in (**F**). (**H**) The western blot analysis of Bcl-xL, Bax, and Bcl-2 protein expression. (**I**–**K**) The densitometric semi-quantifications of the western blots in (**H**). Means with different small letters were significantly different (*p* < 0.05).

The apoptosis rate of PC12 cells was measured using a BD Accuri^TM^ C6 Flow Cytometer shown in Figure 5A,B. The total apoptotic cells were composed of the early (Q3) and the late (Q2) apoptotic cells. The total cell apoptosis rate increased dramatically due to CoCl_2_ exposure, which was 10.07 times that in the untreated control. In other words, CoCl_2_-induced hypoxic damage significantly induced apoptosis in PC12 cells. In contrast, treatment of PC12 cells in hypoxic stress with UT extracts at 2 mg/mL resulted in a remarkable inhibition of the apoptotic response by 52.87%. Additionally, a minimal apoptosis rate was observed in FT-treated cells, which was 59.39% lower than that in the UT-treated cells.

To further study the protective mechanisms of UT and FT, the cell apoptosis pathway-related proteins, including caspase-3, caspase-8, caspase-9, Bax, Bcl-2, and Bcl-xL were investigated as well. Figure 5C–G showed that CoCl_2_ treatment significantly up-regulated caspase-3, caspase-9, and caspase-8, the three pro-apoptotic proteins, by degrading pro-caspase-3, 9, and 8 into their active state. The cleaved-caspase-3 cleaved-caspase-9 and cleaved-caspase-8 protein levels in only CoCl_2_-treated cells significantly increased by 81.44%, 16.98%, and 133.53% compared to control. However, significant decreases were obtained in the cleaved-caspase-3, cleaved-caspase-9, and cleaved-caspase-8 protein levels after UT or FT treatments. For example, the cleaved-caspase-3, cleaved-caspase-9, and cleaved-caspase-8 activities in FT-treated cells were only 70.88%, 75.34%, and 37.41% of those in the hypoxic control, respectively. A similar result was also observed in pro-apoptotic protein Bax (Figure 5K). Inversely, the induced decrease by CoCl_2_ in anti-apoptotic proteins Bcl-2 and Bcl-xL was significantly inhibited after UT or FT treatments (Figure 5H–J) where the Bcl-2 and Bcl-xL protein levels in FT-treated cells respectively increased by 71.43% and 31.58% (*p* < 0.05). Therefore, FT effectively protected PC12 cells against the hypoxic injury-induced apoptosis through up-regulation of anti-apoptotic proteins Bcl-2 and Bcl-xL and down-regulation of pro-apoptotic protein Bax, caspase-3, caspase-8, and caspase-9. Cell apoptosis is a complex procedure regulated largely by pro-apoptosis factors acting for or against the final death event [33]. The culmination of the balancing act between these factors at signal transduction checkpoints may result in the final decision to die or live. Therefore, the apoptosis induced by CoCl_2_ is the result of the cooperation of many pro- and anti-apoptosis factors, which explained the reason why the western blots of caspase-9, caspase-8, caspase-3, Bcl-xL, Bax, and Bcl-2 were not in proportion to the increased apoptosis rate of PC12. Shi et al. (2019) showed that soy genistein attenuated CoCl_2_-induced cell death in H9c2 cardiomyocytes by up-regulating the Bcl-2/Bax ratio and down-regulating caspase-3 expression [34]. Qi et al. (2017) indicated that tea polyphenols exhibit neuroprotective action against oxidative stress-induced apoptosis in SH-SY5Y cells through the activation of the Keap1/Nrf2 signaling pathway and TrkB/CREB/BDNF pathway [28]. Akinrinde and Adebiyi (2019) also found that gallic acid could protect Wistar rats against CoCl_2_-induced toxicity by restoration of antioxidant enzyme activities and Ca^2+^ homeostasis, as well as inhibition of oxidative stress in the brain [31]. Hence, the observed anti-apoptotic effect of FT could be attributed to its high phenolic content, especially isoflavones.

### 2.6. Inhibitory Effects of UT and FT on Cell Cycle in Hypoxic Injury

**Figure 6:**
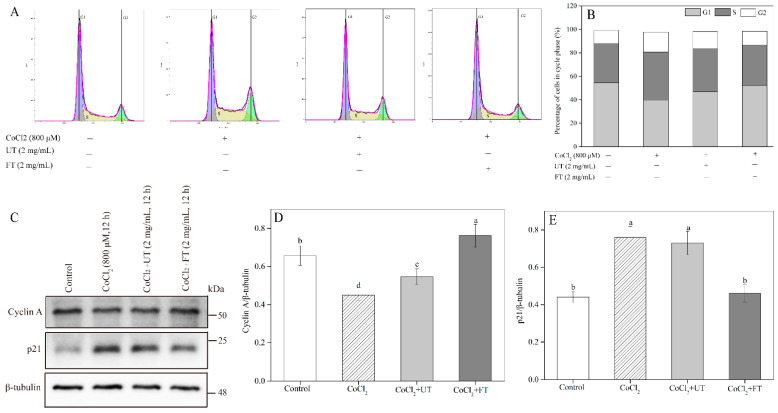
Effects of UT and FT extract on the CoCl_2_-induced cell cycle arrest. (**A**) Cell cycle measured using a BD Accuri^TM^ C6 Flow cytometer. (**B**) The densitometric semi-quantifications of the western blots in (**A**). (**C**) The western blot analysis of cyclin A and p21 protein expression. (**D**,**E**) The densitometric semi-quantifications of the western blots in (**C**). Means with different small letters were significantly different (*p* < 0.05).

Profound cell cycle alterations can result in cell death [35]. As shown in Figure 6A,B, an obvious S-phase cell cycle arrest was observed in CoCl_2_-treated cells. The cell number at the S phase significantly increased by 21.47% and the cell number at the G1 phase decreased by 26.84% compared to control. The S-phase cell cycle arrest induced by CoCl_2_ was significantly inhibited by UT and FT treatments. For example, the cell number at the S phase decreased by 16.21% and the G1 phase cell number increased by 31.16% in FT extract-treated group compared with those in hypoxic control. The crucial proteins involved in the cell cycle, including cyclin A and p21, were measured to study the cellular mechanisms (Figure 6C–E). The cyclin A protein level in PC12 cells suffered a significant decrease after CoCl_2_ treatment, which reduced by 31.51% compared to control. At the same time, a significant increase (72.34%) in the p21 protein level was observed in the CoCl_2_-treated group. The observed S-phase cell cycle arrest induced by CoCl_2_ was significantly attenuated by UT or FT extracts through significant up-regulation of the cyclin A protein level and down-regulation of the p21 protein level in PC12 cells. FT exhibited a more significant inhibitory effect on CoCl_2_-induced S-phase arrest than UT through improving a higher expression level of cyclin A protein and limiting p21 protein expression.

### 2.7. Activation of Cell Autophagy by the UT and FT

**Figure 7:**
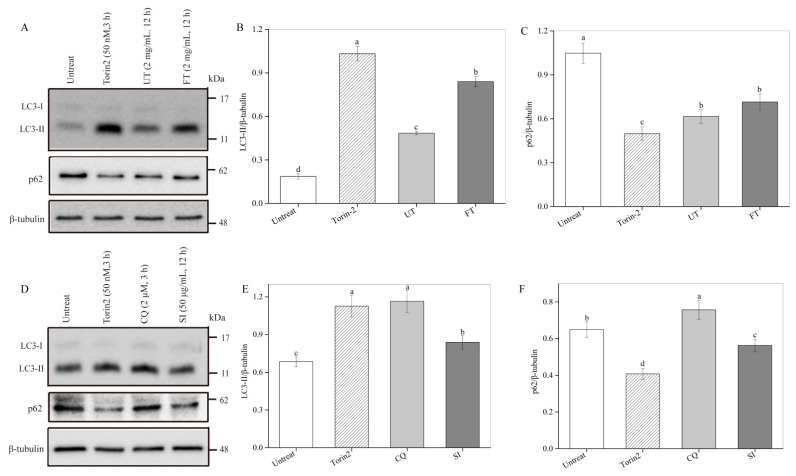
Effects of UT and FT extract on autophagy in PC12 cells. (**A**) The western blot analysis of p62 and protein LC3-II expression in PC12 cells treated with UT and FT extract. (**B**,**C**) The densitometric semi-quantifications of the western blots in (**A**). (**D**) The western blot analysis of p62 and protein LC3-II expression in PC12 cells treated with SI. (**E**,**F**) The densitometric semi-quantifications of the western blots in (**D**). SI, soy isoflavone extract with 80% purity. Means with different small letters were significantly different (*p* < 0.05).

Intracellular autophagy can play an important role in chemical hypoxia- and oxidant-induced damage [36]. To monitor autophagy, the alterations of two marker proteins, LC3-II and p62, were analyzed by western blot (Figure 7) [30]. Torin-2 is a novel, second-generation ATP-competitive inhibitor that is potent and selective for mTOR [37]. In the present study, Torin2-treated cells were used as a positive control. As shown in Figure 7A–C, UT and FT extract treatment significantly increased the LC3-II protein level compared with the untreated control. The LC3II/β-tubulin ratio in UT and FT-treated cells was respectively 2.61 and 4.52 times that in the untreated control, while the p62 protein level in UT or FT extract-treated cells exhibited a significant decrease, which was respectively 58.78% and 68.23% of that in the untreated control. LC3-II level is well correlated with the intracellular autophagosome number, but the p62 level is negatively correlated with the autophagy level in cells [38,39]. Autophagy can also reduce ROS-induced oxidative injury by degradation of damaged organelles and protein aggregates [40,41]. Therefore, tofu extract treatment and, in particular, FT treatment may have significantly activate autophagy in PC12 cells and, as a result, enhanced the survival of PC12 cells in hypoxic injury.

**Figure 8:**
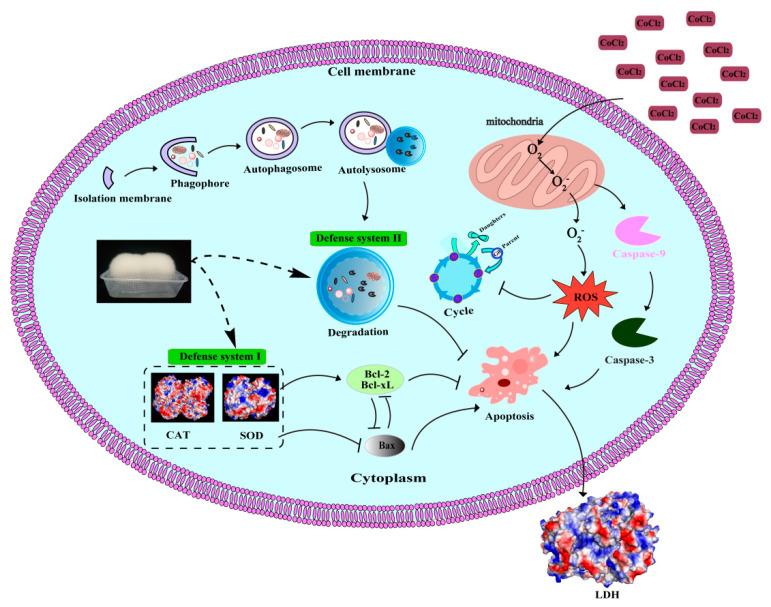
Schematic diagram of the potential mechanisms of the neuroprotective effects of FT in vitro.

As shown in Table 1, a high phenolic content, especially isoflavones, was obtained in UT and FT extracts. To confirm the contribution of isoflavones in autophagy, an isoflavone extract with 80% purity was also tested (Figure 7D–F). The isoflavone treatment significantly increased the LC3-II level by 22.16% (*p* < 0.05) and decreased the p62 level (*p* < 0.05) in PC12 cells compared to control. Previous studies reported similar results. Li et al. (2017) indicated that isoflavones can activate BEX2-dependent autophagy to protect against Atrazine-induced neuronal apoptosis in SH-SY5Y cells [42]. Liu et al. (2019) found that quercetin-modified gold-palladium nanoparticles can efficiently induce autophagy and promote the fusion of lysosomes and autophagosomes to protect SH-SY5Y cells against Aβ-induced cytotoxicity injury [22]. Cheng et al. (2019) demonstrated that treatment with ferulic acid exhibits neuroprotective effects by up-regulating HSP70/autophagy and HSP70/Bcl-2-induced signaling pathways [23]. Tong et al. (2012) reported that ghrelin-induced autophagy plays an important role in protecting H9c2 cells against hypoxic injury induced by CoCl_2_ [43]. Therefore, the neuroprotective effects of UT and FT might be partly owed to the autophagy induced by their phenolics. As summarized in Figure 8, FT had the potential to protect cells against hypoxic damage in vitro through the following pathways: (1) enhancing the intracellular antioxidant mechanism such as SOD and CAT to eliminate the excessive ROS, (2) activating autophagy to degrade the damaged organelles and protein aggregates, (3) inhibiting cell apoptosis through up-regulation of anti-apoptotic proteins Bcl-2 and Bcl-xL and down-regulation of pro-apoptotic protein Bax, caspase-3 and caspase-9, and (4) attenuating S-phase cell cycle arrest by up-regulation of the cyclin A and down-regulation of the p21 protein in hypoxic injury. A past report showed that soybean isoflavones can quickly cross the blood-brain barrier in SD rats that suffered two surgeries for implanting cannulas at lateral ventricle and right jugular vein for brain microdialysis and blood collection, respectively [44], making it a potential drug for treating neurodegenerative diseases. Therefore, the neuroprotective effects of the FT extract will be further studied in mice.

## 3. Materials and Methods

### 3.1. Materials and Microorganism

The soybean used in this study was purchased from a local market. The isoflavone extract (80% purity) and high purity standards of chlorogenic acid, vanillic acid, caffeic acid, daidzin, glycitin, ferulic acid, genistin, daidzein, glycitein, quercetin, and genistein were purchased from Shanghai Yuanye Bio-Technology Co., Ltd. (Shanghai, China). The neuron-like rat pheochromocytoma cell lines (PC12) were obtained from Nanjing Keygen Biotech. Co., Ltd. (Nanjing, China). Penicillin/streptomycin, fetal bovine serum (FBS), and Dulbecco’s modified Eagle’s medium (DMEM), were purchased from Gibco (CA, USA). The kits for LDH (C0016), ROS (S0033S), MDA (S0131), SOD (S0109), CAT (S0051), cell apoptosis (C1062M), protein content (P0012S), and cell cycle (C1052) were purchased from Shanghai Beyotime Institute of Biotechnology (Shanghai, China). Antibodies for p21, cyclin A, Bcl-2, Bax, Bcl-xL, LC3-II, p62, caspase-3, caspase-8, caspase-9, and β-tubulin, were obtained from Shanghai Abmart Inc (Shanghai, China). CoCl_2_ was purchased from Kermel Chemical Reagent Co. Ltd. (Tianjin, China). All other used reagents were of analytical grade.

The *A. elegans* (GenBank MT229136) used in this study was isolated from a commercial *Mao-tofu*, a fermented soybean food from the Huangshan region (Anhui Province, China). The strain was cultured on a potato dextrose agar at 25 °C for 4 days to form spores. The spores were scraped and diluted with sterile distilled water to a final concentration of 1 × 10^6^ spores/mL.

### 3.2. Tofu Fermentation and Sample Preparation

FT was prepared using the method provided in our previous study [18]. Shortly, 1 kg clean soybeans were soaked in water for 10 h and then grounded with 6 L of water. The mixture was filtered to obtain soymilk. The milk was incubated at 95 °C for 10 min. After the temperature cooled to 85 °C, MgCl_2_ solution (0.3% *w*/*v*) was added in milk. The proteins were coagulated for 20 min and then moved to a tofu cast. The tofu was generated after being pressed for about 10 min and then cut into pieces for fermentation. The tofu pieces were sterilized at 115 °C for 20 min. Afterwards, 0.2 mL of spore suspension (1 × 10^6^ spores/mL) was inoculated at the surface of 10 g tofu and fermentation was conducted at 22 °C for 5 d to obtain FT. FT was freeze-dried and milled into powder. Sample powder was extracted with 80% methanol and centrifuged at 10,000× *g* for 20 min. The residue was re-extracted twice. The combined supernatant was concentrated, and the lipid was removed by hexane. Then the supernatant was extracted with ethyl acetate and freeze-dried for further study. In the control group, the UT pieces were sterilized at 115 °C for 20 min, inoculated with an equal amount of sterile water without spores, and kept at 22 °C for 5 d. Then UT was extracted under the same conditions with FT and freeze-dried.

### 3.3. Analysis for Phenolic Content and Antioxidant Activity

The sample was dissolved in 80% methanol (*v*/*v*) and filtered using a 0.22 μm syringe filter. The phenolic composition was analyzed using HPLC. The mobile phases were composed of buffer A (0.4% acetic acid in water) and B (acetonitrile). Elution was conducted using the following gradient: 5–25% buffer B, 0–40 min; 2–35% buffer B, 40–45 min; 35–50% buffer B, 45–50 min; 50–5% buffer B, 50–55 min. The flow rate and detection wavelengths were 0.8 mL/min and 254 nm, respectively. The TPC and antioxidant activity, including the ABTS radical cation scavenging capacity, RP, and FRAP, was measured following the method developed by Xiao et al. (2014) [7].

### 3.4. Cell Culture

PC12 cells were inoculated in a 25 cm^2^ tissue culture flask in DMEM supplemented with FBS (10%), penicillin (100 U/mL), and streptomycin (100 μg/mL). Cells were incubated in a humidified atmosphere of 5% CO_2_ at 37 °C for two days before use.

### 3.5. Cytotoxicity Assay

Cell viability was determined by MTT assay [30]. Briefly, cells were inoculated in 96-well plates at a density of 1.4 × 10^4^ cells/well for 12 h and then exposed to CoCl_2_ (100–1000 μM) in the absence or presence of the UT or FT extracts (0.125–4 mg/mL) for 12 h. The MTT solution was added at a final concentration of 1 mg/mL, and plates were incubated for another 4 h. After the culture medium was removed, 150 μL of dimethylsulfoxide (DMSO) was added and then the absorbance was read at 490 nm.

### 3.6. Cell Viability Assay

PC12 cells were inoculated in 96-well plates at a density of 1.4 × 10^4^ cells/well for 12 h. Then, cells were treated with 800 μM CoCl_2_ for 12 h to cause hypoxic injury and incubated with or without UT or FT extracts (0.25–2 mg/mL) at the same time. After treatment, the cell viability was measured by the MTT assay described above. The cell morphology under different treatments was monitored using microscopy (ECLIPSE TE2000-S, Nikon, Japan).

### 3.7. Effects of UT and FT on the LDH Release under Hypoxic Injury

PC12 cells were cultured on a 96-well plate at a density of 1.4 × 10^4^ cells/well at 37 °C for 12 h. Then the cells were treated with 800 μM CoCl_2_ for 12 h in the absence or presence of the UT or FT extracts (0.25–2 mg/mL) at the same time. The LDH level was determined using an LDH assay kit according to the manufacturer’s description. The data were presented as the percentage of the untreated control.

### 3.8. Effects of UT and FT on ROS Formation during Hypoxic Injury

PC12 cells were cultured on a 6-well plate at a density of 2.8 × 10^5^ cells/well at 37 °C for 12 h. Then cells were treated with 800 μM CoCl_2_ for 12 h in the absence or presence of the UT or FT extracts (2 mg/mL) at the same time. The ROS levels of PC12 cells were determined using a ROS assay kit according to the manufacturer’s instruction. PC12 cells were washed and treated with 10 μM DCFH-DA at 37 °C for 20 min. Then cells were washed thrice with a serum-free cell culture medium. The fluorescence intensity was determined using a BD Accuri^TM^ C6 Flow cytometer. Results were presented as the percentage of the untreated control.

### 3.9. Effects of UT and FT on the MDA Level under Hypoxic Injury

PC12 cells were cultured on a 6-well plate at a density of 2.8 × 10^5^ cells/well at 37 °C for 12 h. Then the cells were treated with 800 μM CoCl_2_ for 12 h in the absence or presence of the UT or FT extracts (0.25–2 mg/mL) at the same time. The MDA level was determined using an MDA assay kit according to the manufacturer’s description. Briefly, the adherent cells were separated by trypsin, washed twice with PBS, and broken using an ultrasonic cell disruptor. The supernatant was obtained after centrifugation at 10,000× *g* and 4 °C for 20 min. Then, 0.1 mL of the supernatant was mixed with 0.2 mL of MDA working solution and then maintained at 100 °C for 15 min. After cooling down to room temperature, the absorbance at 532 nm was read. The data were presented as the percentage of the untreated control.

### 3.10. Effects of UT and FT on Antioxidant Enzyme Activities during Hypoxic Injury

PC12 cells were treated as above. The adherent cells were separated by trypsin, washed twice with PBS, and broken using an ultrasonic cell disruptor. The supernatant was used to measure the antioxidant enzyme activities after centrifugation at 10,000× *g* and 4 °C for 20 min. The SOD and CAT activities were assessed using corresponding assay kits according to the manufacturer’s instruction. Briefly, the supernatant was mixed with enough hydrogen peroxide (H_2_O_2_). The remained H_2_O_2_ oxidized a phenol substrate into a red quinoneimine product with maximum absorption at 520 nm. The CAT activity was negatively correlative to 520 nm absorption. One unit of CAT activity was defined as the amount required to catalyze 1 μmol H_2_O_2_ per min at 25 °C and pH 7.5. The total SOD activity was determined by scavenging of O_2_− generated by xanthine. The remained O_2_- oxidized nitroblue tetrazolium (NBT) dye to NBT formazan with maximum absorption at 560 nm. One unit of SOD activity was defined as the amount required to inhibit the rate of NBT reduction by 50% at 37 °C during 30 min. The protein content in the supernatant was measured using a bicinchoninic acid (BCA) protein assay kit.

### 3.11. Effects of UT and FT on Cell Apoptosis during Hypoxic Injury

PC12 cells were treated as above. The adherent cells were harvested by trypsin and treated according to the description of the cell apoptosis kit. Briefly, PC12 cells were washed with cell culture medium and then re-suspended in 195 μL Annexin V-FITC Binding Buffer. Then, 5 μL Annexin V-FITC and 10 μL prodium iodide (PI) were added. The mixture was maintained at room temperature for 20 min. The fluorescence intensity was measured using a BD Accuri^TM^ C6 Flow cytometer within 1 h. The cell apoptosis-related proteins, including Bax, Bcl-2, caspases-3, caspases-8, caspases-9, and Bcl-xL, were analyzed using western blot.

### 3.12. Effects of UT and FT on Cell Cycle during Hypoxic Injury

PC12 cells were treated as above. The adherent cells were harvested by trypsin and treated according to the description of the cell cycle kit. Shortly, PC12 cells were washed using cold PBS and fixed in ethanol (70%, *v*/*v*) at 4 °C for 30 min. Cells were rewashed and suspended in PI staining reagent. The mixture was kept at 37 °C in the dark for 30 min. The fluorescence intensity was measured using a BD Accuri^TM^ C6 Flow cytometer within 1 h. The cell cycle-related proteins, including cyclin A and p21, were analyzed using western blot.

### 3.13. Effects of UT and FT on Autophagy

PC12 cells were cultured on a 6-well plate at a density of 2.8 × 10^5^ cells/well at 37 °C for 12 h. Then cells were exposed to UT or FT extracts (2 mg/mL) for 12 h at the same time. The adherent cells were lysed in RIPA buffer and then centrifuged at 10,000× *g* and 4 °C for 10 min. The autophagy pathway proteins, including LC3 and p62, were analyzed using western blot.

### 3.14. Western Blot Analysis

The western blot analysis was performed following Zhang et al. (2020) [30]. Shortly, PC12 cells were lysed in RIPA buffer composed of 50 mM Tris (pH 7.4), 150 mM NaCl, 1% NP-40, 0.5% sodium deoxycholate, 0.1% sodium dodecyl sulfate, and several inhibitors such as sodium orthovanadate and sodium fluoride. The lysate was centrifuged at 10,000× *g* and 4 °C for 10 min. The protein content in the supernatant was measured using a BCA protein assay kit. Proteins (30 μg/lane) of the cell lysates were separated by 10% or 15% sodium dodecyl sulfate-polyacrylamide gel electrophoresis (SDS-PAGE) and transferred to a 0.22 μm polyvinylidene fluoride membrane (Bio-Rad, USA). The membrane was exposed to antibodies against Bcl-2, cyclin A, p21, caspases-9, caspases-8, caspases-3, Bcl-xL, Bax, LC3, p62, and β-tubulin, respectively. The protein signals were enhanced using horseradish peroxidase (HRP)-conjugated secondary antibody and measured using an ECL Western Blot Detection System (GE ImageQuant LAS4000mini, USA). The densitometric semi-quantifications of the western blot results were determined using ImageJ software.

### 3.15. Statistical Analysis

All data were analyzed by applying variance (ANOVA) multiple comparisons (one way) in SPSS and expressed as mean ± standard deviation (SD). The difference significance was determined using the Duncan test. For all statistical tests, *p* < 0.05 was considered as the significance level.

## 4. Conclusions

The present study indicated that *A. elegans* fermentation significantly improved the biological activity of tofu. The TPC of tofu significantly increased and glucoside isoflavones were converted to their aglycone form during fermentation. The FT extract displayed high neuroprotective effects against CoCl_2_-induced hypoxic injury by accelerating cell proliferation, maintaining cell morphology, reducing LDH leakage, inhibiting ROS formation, reducing the MDA level, and promoting antioxidant enzyme activities. Additionally, CoCl_2_-induced cell apoptosis and cell cycle arrest were significantly attenuated by FT extract. The results strongly suggested that fermented tofu with *A. elegans* as a functional soybean food has a high potential to improve consumers’ health by protecting nerve cells against hypoxic injury.

## Figures and Tables

**Table 1:** The phenolic content of UT and FT extracts.

Peak	Compounds	UT (mg/100 g)	FT (mg/100 g)
1	Chlorogenic acid	1.08 ± 0.11 ^F,a^	0.83 ± 0.08 ^C,b^
2	Vanillic acid	7.87 ± 0.41 ^F^	N.D.
3	Caffeic acid	N.D.	N.D.
4	Daidzin	361.90 ± 27.49 ^A,a^	20.86 ± 2.39 ^C,b^
5	Glycitin	93.24 ± 3.63 ^E,a^	7.30 ± 0.75 ^C,b^
6	Ferulic acid	N.D.	N.D.
7	Genistin	207.47 ± 22.80 ^B,a^	30.84 ± 1.72 ^C,b^
8	Daidzein	106.71 ± 13.48 ^D,E,b^	301.16 ± 21.81 ^B,a^
9	Glycitein	174.39 ± 12.89 ^C,a^	423.69 ± 31.13 ^A,b^
10	Quercetin	N.D.	N.D.
11	Genistein	127.51 ± 17.40 ^D,b^	422.97 ± 31.85 ^A,a^
	TPC (GAE mg/100 g)	1264.69 ± 54.08 ^b^	1835.67 ± 78.55 ^a^

* *p* < 0.05 was considered as the significant level. The small letters a and b were used to show the significant difference between UT and FT for the same component where “^a^” indicates a significantly higher yield than “^b^”. The capital letters (^A^, ^B^, ^C^, ^D^, ^E^, and ^F^) within UT indicate the significant difference and the ordering of results, since A means the highest yield and F means the smallest amount. The capital letters (^A^, ^B^, and ^C^) within FT indicate the significant difference and the ordering of results, since A means the highest yield and C means the smallest amount. UT, unfermented tofu; FT, fermented tofu. N.D., not detected.

**Table 2:** The antioxidant activities of UT and FT extracts.

Samples	ABTS^+^ Scavenging Activity (%)	RP (OD_700_)	FRAP (μM FeSO_4_)
UT	67.50 ± 3.17 ^b^	0.18 ± 0.02 ^b^	380.54 ± 18.23 ^b^
FT	89.97 ± 1.20 ^a^	0.33 ± 0.02 ^a^	643.08 ± 20.28 ^a^

* *p* < 0.05 was considered as the significant level. The small letters a and b were used to show the significant difference between UT and FT for the same antioxidant assay where “^a^” indicates a significantly higher yield than “^b^”. UT, unfermented tofu; FT, fermented tofu.

## Data Availability

The data presented in this study are available on request from the corresponding author.
